# Retraction: Efficacy of Holmium: Yttrium-Aluminum-Garnet (YAG) Laser in the Treatment of Proximal Ureteral Stones in Adults

**DOI:** 10.7759/cureus.r199

**Published:** 2025-11-11

**Authors:** Zafar Ahmad Khan, Javed Miandad, Rizwan Kundi, Komal Amin, Mehran Fazal, Miraj Ahmad

**Affiliations:** 1 Department of Urology, Medical Teaching Institution, Mardan Medical Complex, Mardan, PAK; 2 Department of Urology, Sindh Institute of Urology and Transplantation, Karachi, PAK; 3 Department of Physiology, Bacha Khan Medical College, Mardan, PAK

This article has been retracted by the Editors-in-Chief due to the presence of severe flaws, including unreliable statistical analyses, missing data, and inconsistencies between the methods, results, and discussion. The authors disagree with the decision to retract.

